# TNFR2 Expression on CD25^hi^FOXP3^+^ T Cells Induced upon TCR Stimulation of CD4 T Cells Identifies Maximal Cytokine-Producing Effectors

**DOI:** 10.3389/fimmu.2013.00233

**Published:** 2013-08-06

**Authors:** Chindu Govindaraj, Karen Scalzo-Inguanti, Anja Scholzen, Shuo Li, Magdalena Plebanski

**Affiliations:** ^1^Department of Immunology, Monash University, Clayton, VIC, Australia; ^2^Department of Microbiology and Immunology, The University of Melbourne, Clayton, VIC, Australia

**Keywords:** regulatory T cells, FOXP3, TNFR2, Th1, effector cells

## Abstract

In this study, we show that CD25^hi^TNFR2^+^ cells can be rapidly generated *in vitro* from circulating CD4 lymphocytes by polyclonal stimuli anti-CD3 in the presence of anti-CD28. The *in vitro* induced CD25^hi^TNFR2^+^ T cells express a conventional regulatory T cells phenotype FOXP3^+^CTLA4^+^CD127^lo/−^, but produce effector and immunoregulatory cytokines including IL-2, IL-10, and IFN-g. These induced CD25^hi^TNFR2^+^ T cells do not suppress target cell proliferation, but enhance it instead. Thus the CD25^hi^TNFR2^+^ phenotype induced rapidly following CD3/28 cross linking of CD4 T cells identifies cells with maximal proliferative and effector cytokine-producing capability. The *in vivo* counterpart of this cell population may play an important role in immune response initiation.

## Introduction

Regulatory T cells (T_regs_) play a central role in the maintenance of peripheral tolerance and immune homeostasis, thereby preventing autoimmune diseases (1–3). FOXP3 is a key transcription factor for T_regs_ (4–6), with ectopic expression of FOXP3 in human CD4^+^ T cells resulting in the acquisition of suppressive function and down-regulation of effector cytokine production like IFN-g (4, 5, 7). Although all murine FOXP3^+^ T cells are regulatory in function, the definition of human T_regs_ using FOXP3 is complicated by the fact that effector T cells up-regulate FOXP3 expression upon activation (8). FOXP3 expression on activated effector T cells has however been reported to be transient and relatively low when compared to T_regs_ (9). Such low levels are believed to be insufficient to negatively regulate effector cytokine production, particularly IFN-g (8). This suggests that T cells that are FOXP3^hi^ are regulatory in function. However, FOXP3 is an intracellular transcription factor and functional assays cannot be performed based on FOXP3 expression in human T cells. Hence, a surrogate marker that is expressed on the surface of T_regs_ is required to distinguish bona fide T_regs_.

Recent studies have identified a subset within both murine and human T_regs_ that expresses the type II receptor for the major pro-inflammatory cytokine tumor necrosis factor TNF, TNFR2 (1, 10, 11). As well as providing a potential link between the regulation of inflammation and adaptive immunity, *ex vivo* TNFR2^+^ T_regs_ are maximally suppressive regulators in both mice and humans, consistent with their higher expression of CTLA4 and FOXP3 (12–14). Additionally, intracellular FOXP3 expression appeared to positively correlate with surface TNFR2 expression on human CD4 T cells (1). However, similar to FOXP3 expression, both murine and human effector T cells also up-regulate TNFR2 expression upon activation via the T cell receptor (TCR) (15, 16). A recent murine study demonstrates FOXP3^−^TNFR2^+^ effector T cells secrete significantly higher levels of Th1 cytokines like IFN-g when compared to FOXP3^−^TNFR2^−^ effector T cells (17). These effector T cells, however, are in turn susceptible to suppression exerted by TNFR2^+^FOXP3^+^ T_regs_ (17). The above data suggest that TNFR2 expression identifies the maximally functional effector T cells (CD25^int^TNFR2^+^FOXP3^int^) and T_regs_ (CD25^hi^TNFR2^+^FOXP3^hi^) in humans. We hypothesized that human CD25^hi^ T cells expressing TNFR2 identifies T_regs_ and TNFR2 may be a surrogate marker for FOXP3.

Herein we show that although human CD25^hi^TNFR2^+^FOXP3^hi^ T cells with a T_reg_ phenotype are inducible *in vitro* from isolated CD4 T cells by stimulation via the TCR, these induced cells fail to suppress proliferation of effector cells, and are surprisingly the maximally effector cytokine-producing population, capable of augmenting early proliferative responses.

## Materials and Methods

### Cell isolation

Peripheral blood mononuclear cells (PBMCs) were isolated from buffy coats of healthy individuals, provided by the Australian Red Cross Blood Service. CD4^+^ T cells were isolated from PBMCs using the CD4 T cell negative isolation kit and LD columns according to manufacture’s recommendations (Miltenyi Biotec). The purified fraction consistently contained 94–99% CD3^+^CD4^+^ T cells by flow cytometry. CD4^+^CD25^−^ and CD4^+^CD25^+^ cells were obtained by staining CD4 cells with anti-CD25 PE antibody and anti-PE magnetic cell isolation beads as per manufacture’s protocol (BD Pharmingen).

### *In vitro* induction of CD25^hi^TNFR2^+^ cells

To obtain the induced TNFR2^+^ T cell subsets, the MACS purified T cell populations, either un-fractionated CD4^+^ T cells, or its sub-populations, CD4^+^CD25^−^ and CD4^+^CD25^+^ T cells were cultured. The T cells were suspended in AIM V medium (Invitrogen) containing 5% heat inactivated normal human serum (Sigma). The cells were added (5 × 10^6^ cells/2 mL/well) to 24 well plates, pre-coated with anti-CD3 antibody (2.5 μg/mL; OKT3, Biolegend). This was followed by the addition of soluble anti-CD28 antibody (1.25 μg/mL; CD28.2, BD Pharmingen), and the cells were cultured for 72 h at 37°C with 5% CO_2_.

### Cell sorting

The above un-fractionated CD4^+^ T cell culture was harvested on day 3, and sorted using a FACS ARIA (Becton Dickinson) to isolate the CD25^hi^TNFR2^+^, CD25^int^TNFR2^int/−^ and CD25^−^TNFR2^−^ T cell populations.

### Flow cytometry

The following monoclonal antibodies were used for flow cytometry analysis: TNFR2 FITC (R&D systems), CD3 FITC/APC, CD4 APC-Cy7, CD25 PE/PeCy7, CD127 bio-PerCP, CTLA4 APC (BD Pharmingen), and FOXP3 APC/PerCP. Intracellular staining was performed by firstly using the FOXP3 fixation/permeabilization kit (eBioscience) followed by staining the cells intracellular using the FOXP3 antibody. Flow cytometry was performed using BD LSRII, and data were analyzed using FlowJo software (Treestar).

For intracellular cytokine staining, the MACS isolated total CD4^+^ T cells were cultured for 3 days, stimulating with CD3/28. On day 3, PMA (50 ng/mL) and Ionomycin (1 mg/mL) were added for 5 h, with Brefeldin A (ebioscience) supplementation for the final 4 h. After stimulation, the cells were stained with intracellular IFN-γ, IL-2, and FOXP3 staining. Flow cytometry was performed using BD ARIA, and data were analyzed using FlowJo.

### Suppression assays

For suppression assays, the sorted cells above were irradiated at 40 Gy for use as suppressors. The responder cells consisted of cryopreserved autologous CD4^+^ T cells that are defrosted and washed. The sorted cell subsets, re-suspended at 10^5^ cells/50 μl in AIM V media containing 5% human serum, were mixed with an equal number of the responder cells. The mixture was then added to a 96 U bottom plate (Becton Dickinson) and stimulated for a further 72 h using CD3/28 stimulation as above. On day 3, cells were pulsed overnight at 37°C with 5 μCi/mL per well of TRK 120 titrated thymidine (Amersham, UK). Cells were then harvested and proliferation was determined by thymidine incorporation, measured by a liquid scintillation counter, Topcount NXT (Packard, USA). In some experiments, autologous CD4 depleted (using anti-CD4 microbeads, Miltenyi Biotec) PBMCs were irradiated at 40 Gy, and used as antigen presenting cells. A mixed lymphocyte reaction (MLR) was also used as responders, where PBMCs of three different donors were cultured together.

### Proliferation assay and cytokine beads array

For proliferation assays, the sorted CD25^hi^TNFR2^+^, CD25^int^TNFR2^int/−^ and CD25^−^TNFR2^−^ cells was re-stimulated for 3 days using CD3/28, pulsed with titrated thymidine on day 3 and analyzed as above. Supernatant was removed prior to thymidine addition for cytokine analysis, where the cytokines present in the supernatant were determined using CBA-flex kits (BD Pharmingen) as per the manufacture’s protocol, and data analyzed using the manufacture’s software.

### RNA isolation and real time RT-PCR

Total RNA was isolated from a minimum of 10^5^ cells of each of the sorted TNFR2 subsets using the RNA isolation kit (Roche, Germany). The first-strand cDNA was synthesized using oligo-dT primers and Superscript II reverse transcriptase (Invitrogen). qPCR for IL-10, TGF-b, IFN-g, T-bet FOXP3, and house keeping control 18SrRNA was performed using commercial primers and SYBR green reagent (Life technologies). PCR was performed using an ABI PRISM 7900 (Applied Biosystems). Results for target genes were normalized to 18SrRNA expression and expressed as fold changes between TNFR2 subsets.

### Statistical analysis

To compare between the induced TNFR2 subsets, one-way ANOVA with Tukey’s multiple comparison test was used if data were normally distributed and Kruskal–Wallis test with Dunn’s multiple comparison test was used if the data were not normally distributed (Graphpad 5.0).

## Results

### CD25^hi^TNFR2^+^T cells induced upon *in vitro* stimulation of CD4 T cells via the TCR have a conventional T_reg_ phenotype

**Figure 1:**
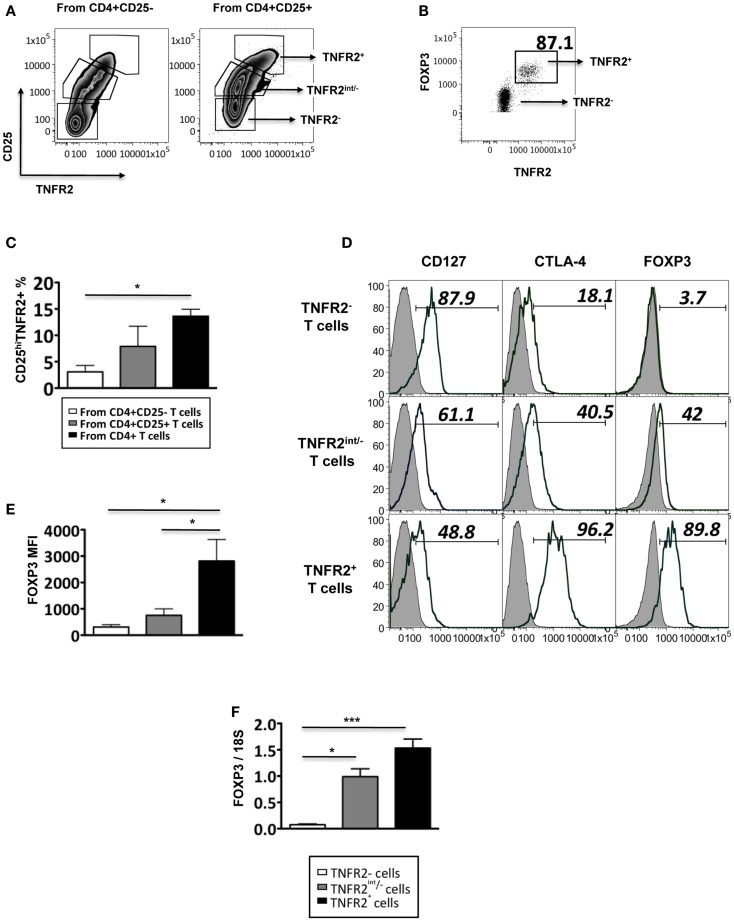
**Phenotype of induced TNFR2^+^ T cells**. **(A)** The expression of CD25 and TNFR2 on MACS sorted CD4^+^CD25^−^ T cells and CD4^+^CD25^+^ cells after 72 h of CD3/28 stimulation. **(B)** The expression of FOXP3 on TNFR2 subsets induced from starting population CD4^+^CD25^+^ T cells. **(C)** Comparison of percentages of CD25^hi^TNFR2^+^ T cells induced from the different starting populations – the CD4^+^CD25^−^, CD4^+^CD25^+^, or un-fractionated CD4^+^ T cells. **(D)** Expression of regulatory molecules on TNFR2 subsets from starting population CD4^+^CD25^+^ T cells. Gray histograms represent isotype staining while clear histogram represents the indicated molecule. The numbers indicate percentage positive for the represented molecular marker. **(E)** The MFI of FOXP3 within the TNFR2 subsets. **(F)** The mRNA expression levels of FOXP3 within the TNFR2 subsets. Data shown in **(A,B,D)** are representative of four donors respectively while **(C,E,F)** are summarized from four donors. Kruskal–Wallis with Dunn’s multiple comparison test was used here and graphs represent mean ± SEM. *p < 0.05.

TNFR2 expression on T_regs_ is believed to be critical for T_reg_ function (12). It is unknown however, if functional TNFR2^+^ T_regs_ can be rapidly generated *in vivo* from circulating human peripheral blood CD4 lymphocytes during an active immune response. To address this question using an *in vitro* model, we purified CD4^+^CD25^−^ T cells and CD4^+^CD25^+^ T cells and stimulated the cells using anti-CD3 in the presence of CD28 to provide the secondary signal. After 72 h, these *in vitro* stimulated T cells could be differentiated into distinct CD25^−^TNFR2^−^, CD25^int^TNFR2^int/−^, and CD25^hi^TNFR2^+^ T cells sub-populations (Figure 1A). While substantial numbers of CD25^hi^TNFR2^+^ T cells were induced from the CD4^+^CD25^−^ and CD4^+^CD25^+^ T cell fractions, we found that these cells were generated more efficiently from the total un-fractionated CD4^+^ T cells (Figure 1C). It is possible that the interactions between CD4^+^CD25^−^ and CD4^+^CD25^+^ cells are helpful for the optimal induction of CD25^hi^TNFR2^+^ T cells under physiological conditions.

**Figure A1:**
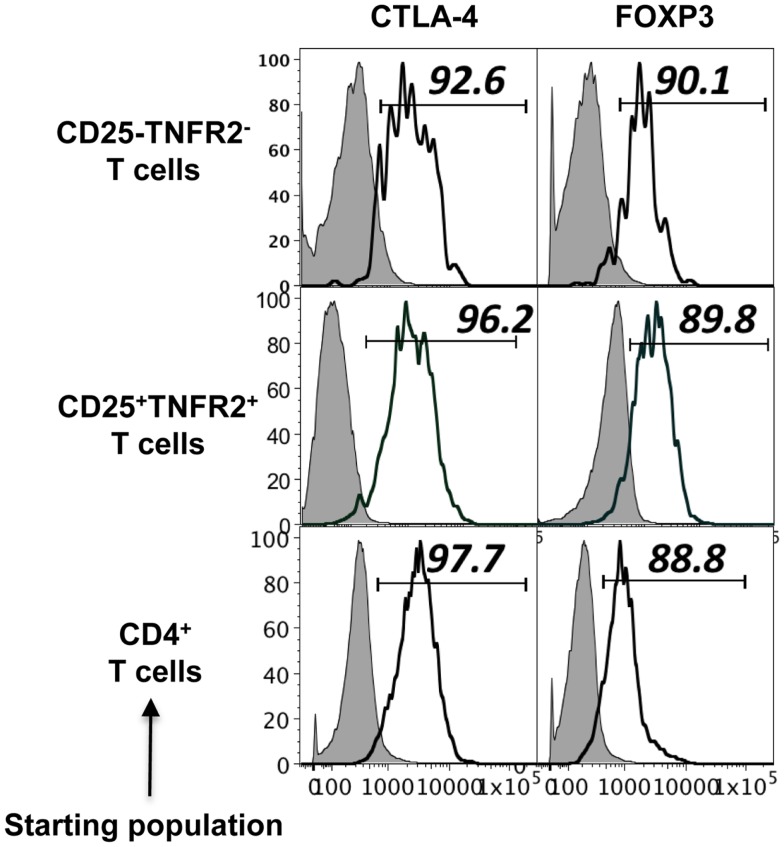
**Phenotype of induced TNFR2^+^ T cells from CD4^+^CD25^−^ T cell, CD4^+^CD25^+^ T cells, and un-fractionated CD4^+^ cells**. Expression of regulatory molecules on TNFR2 subsets from starting population CD4^+^CD25^−^ T cells, CD4^+^CD25^+^ T cells, and un-fractionated CD4^+^ T cells. Gray histograms represent isotype staining while clear histogram represents the indicated molecule. The numbers indicate percentage positive for the represented molecular marker.

These induced CD25^hi^TNFR2^+^ T cells (regardless of the starting population) had a typical T_reg_ phenotype: significantly higher levels of FOXP3, CTLA4, and lower levels of CD127, when compared to the CD25^−^TNFR2^−^ and CD25^int^TNFR2^int/−^ cells within the same culture. Figure 1D is a representative phenotype of CD25^hi^TNFR2^+^ T cells induced from CD4^+^CD25^+^ T cell population. Figure 1E represents the quantitative analysis for FOXP3 expression on the induced TNFR2 subsets. The induced TNFR2^+^ T cell subset had the highest level of FOXP3 expression when compared to the other TNFR2^int/−^ T cell subsets, as shown in both Figures 1B,D. This was further confirmed at the mRNA level using qPCR (Figure 1F). As the phenotype of the induced CD25^hi^TNFR2^+^ T cells contained similar percentage of cells positive for CTLA4 and FOXP3 (see Figure A1 in Appendix) across all starting populations (CD4^+^CD25^−^, CD4^+^CD25^+^, or un-fractioned CD4^+^ T cells), in the following sections we used the un-fractioned CD4 T cells as starting population.

**Figure 2:**
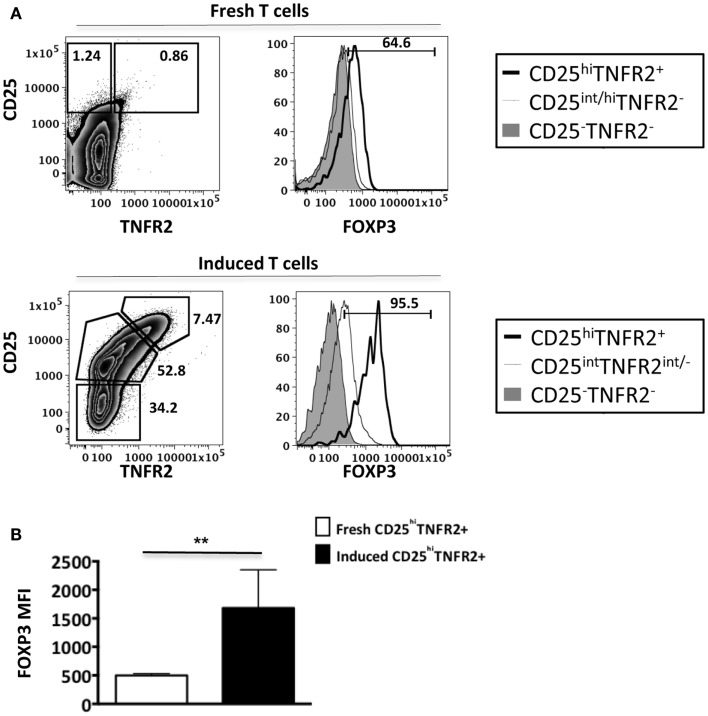
**FOXP3 expression levels on *ex vivo* and induced TNFR2^+^ T cells**. Flow cytometry was performed on both *ex vivo* PBMCs (*N* = 14) and *in vitro* induced T cells (*N* = 4). These cells were initially gated on CD3, CD4, CD25, and TNFR2 expression to identify the different TNFR2 populations, noting that the CD25/TNFR2 phenotype was considerably different between fresh and cultured cells. **(A)** FOXP3 expression was further compared between CD25^−^TNFR2^−^ (tinted histogram), CD25^int^TNFR2^int/−^ (thin clear histogram), and CD25^hi^TNFR2^+^ (thick clear histogram). **(B)** The FOXP3 expression levels were compared between *ex vivo* CD25^hi^TNFR2^+^ and induced CD25^hi^TNFR2^+^ T cells. Unpaired Student’s *t*-test was performed to compare FOXP3 levels and graphs represent mean ± SEM. ****p < 0.0001.

**Figure A2:**
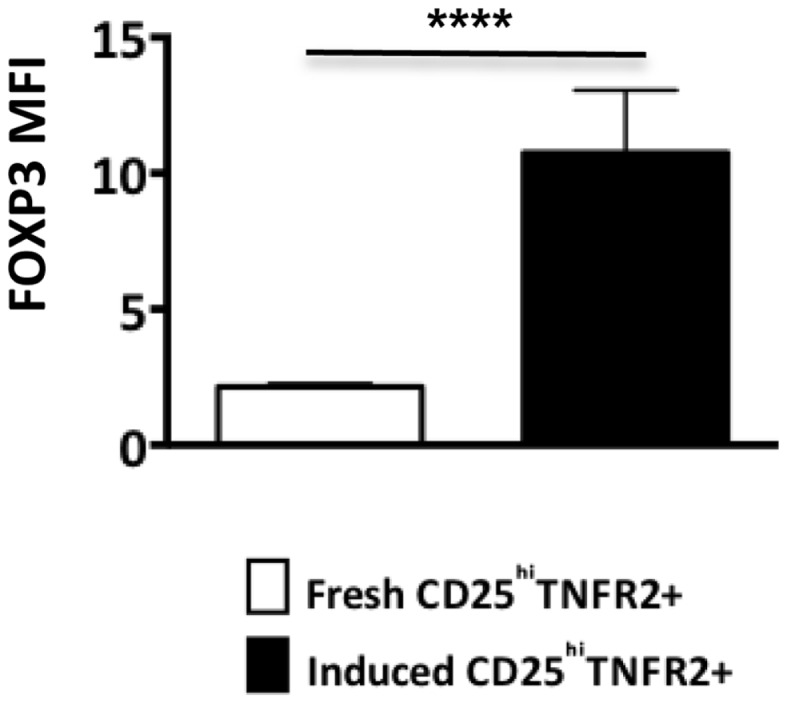
**FOXP3 expression levels on *ex vivo* and induced TNFR2^+^ T cells**. The FOXP3 expression levels were compared between *ex vivo* CD25^hi^TNFR2^+^ and induced CD25^hi^TNFR2^+^ T cells. Unpaired Student’s *t*-test was performed to compare FOXP3 levels and graphs represent mean ± SEM. **p < 0.01.

As conventional effector human T cells also express low levels of FOXP3, it was important to confirm that the *in vitro* CD25^hi^TNFR2^+^ T cells had the highest level of FOXP3 expression. We compared FOXP3 levels between the induced CD25^hi^TNFR2^+^ T cells and *ex vivo* T_regs_ (Figure 2). Firstly, consistent with previous studies, we observed that *ex vivo* T_regs_ expressing TNFR2 had the higher levels of FOXP3 when compared to TNFR2^−^ T_regs_ (Figure 2A). Moreover, induced CD25^hi^TNFR2^+^ T cells had significantly higher levels of FOXP3 when compared to *ex vivo* TNFR2^+^ T_regs_ (Figure 2B). Similar results were obtained even when FOXP3 MFI levels were normalized to the corresponding CD25^−^TNFR2^−^ cells for each donor to avoid any experimental variations and then compared between induced CD25^hi^TNFR2^+^ T cells and *ex vivo* TNFR2^+^ T_regs_ (Figure A2 in Appendix).

Collectively, our data suggests that induced CD25^hi^TNFR2^+^ T cells have a regulatory T cell phenotype and their FOXP3 levels are significantly higher than that of *ex vivo* T_regs_.

### *In vitro* induced CD25^hi^TNFR2^+^ T cells do not suppress proliferative T cell responses

**Figure 3:**
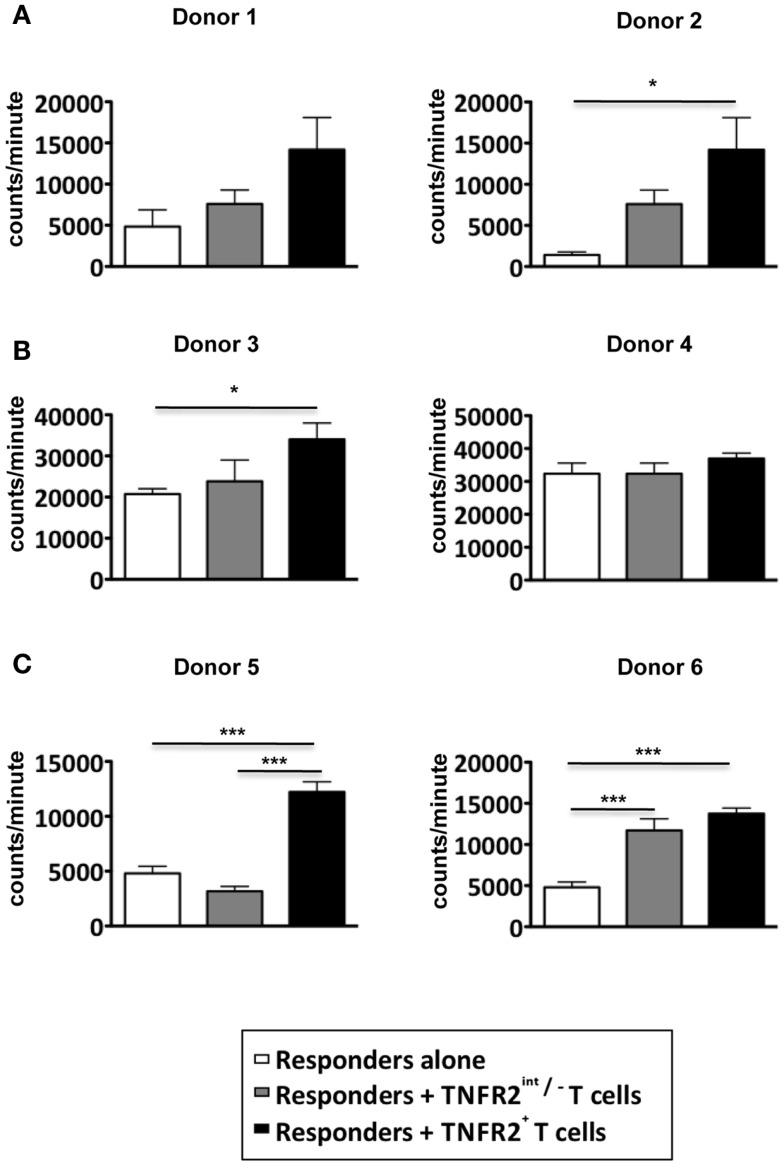
**Suppressive capacity of induced TNFR2^+^ T cells**. CD4^+^ T cells were stimulated with anti-CD3/28 for 72 h. On day 3, induced CD25^int^TNFR2^int/−^ and CD25^hi^TNFR2^+^ were sorted by flow cytometry and added to autologous responders (CD4^+^ T cells) at a ratio of 1:1 and stimulated with anti-CD3/28 for 72 h in the **(A)** absence of APCs or **(B)** presence of APCs. **(C)** Suppression assays performed using MLRs as responders. PBMCs of three different donors were isolated and cultured together with the indicated TNFR2 subsets at a 1:1 ratio. One-way ANOVA with Tukey’s multiple comparison test was used for **(A,C)**, and Kruskal–Wallis with Dunn’s multiple comparison test was used for **(B)**. Error bars indicate SD for **(A,B)** and SEM for **(C)**. Comparison of the proliferation of responders: *p < 0.05; ***p < 0.001.

Since the induced CD25^hi^TNFR2^+^ T cells displayed a typical regulatory T cell phenotype, they would be expected to have regulatory function. Inhibition of proliferative T cell responses is a well-studied suppressor function attributed to T_regs_. The CD25^hi^TNFR2^+^ and CD25^int^TNFR2^int/−^ cells were isolated using flow cytometry on day 3 from the CD4 T cell starting culture, irradiated, and added at 1:1 ratio to responders, which were autologous CD4^+^ T cells. Surprisingly, the induced CD25^hi^TNFR2^+^ T cells did not suppress responder T cell proliferation, but instead, were found to enhance it (Figure 3A). Chen and colleagues demonstrated freshly isolated CD25^+^TNFR2^+^ T cells suppress T cell proliferation in assays that further contain added antigen presenting cells (APCs) (12). We therefore also performed the above suppression assays with the further addition of autologous APCs, to account for any potential indirect suppressor TNFR2^+^ T_reg_ effects. As shown in Figure 3B, the induced CD25^hi^TNFR2^+^ T cells again failed to suppress under these conditions. We speculated that the strong signaling of responder cells by CD3/28 cross linking may not be capable of being suppressed by CD25^hi^TNFR2^+^ T_regs_, but other, more natural T cell stimulation protocols could be susceptible to suppression. The MLR where T cells from donors with different MHC react to each other is a biologically relevant assay (18). We found that induced CD25^hi^TNFR2^+^ T cells were not capable of suppressing MLRs, and instead the addition of these cells into MLR cultures further enhanced proliferative responses (Figure 3C). Therefore, three different independent suppression assays indicated that *in vitro* induced CD25^hi^TNFR2^+^ T cells from healthy CD4 T cells do not have conventional suppressor function.

### Induced CD25^hi^TNFR2^+^ T cells produce effector cytokines IL-2 and IFN-g and are hyper-proliferative

**Figure 4:**
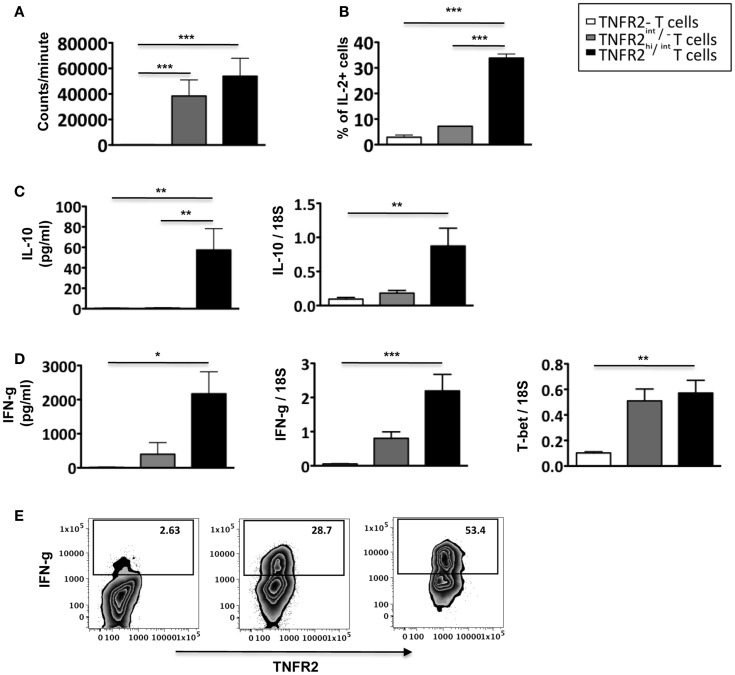
**Proliferative capacity, IL-2, and IFN-g production by induced CD25^hi^TNFR2^+^ T cells**. **(A)** The proliferative capacity of sorted TNFR2 subsets (originated from the un-fractionated CD4^+^ cells) upon 72 h anti-CD3/28 re-stimulation. *N* = 6. **(B)** Intracellular expression of IL-2 (upper panel, *N* = 2) by the sorted cells. **(C)** IL-10 secreted into the supernatant during the re-stimulation of the sorted TNFR2 subsets (*N* = 4) was determined using CBA-flex kits (left panel) and IL-10 mRNA levels (right panel) was determined using qPCR (*N* = 4). **(D)** IFN-g secreted into the supernatant during the re-stimulation of the sorted TNFR2 subsets (*N* = 4) (left panel) and the mRNA expression levels of IFN-g and T-bet on sorted TNFR2 subsets (*N* = 4) (right panel). **(E)** Total PBMCs were stimulated using CD3/28 to obtain the induced TNFR2 populations. On day 3, cells were further stimulated with PMA/Ionomycin in the presence of Brefeldin A to determine intracellular IFN-g production. Flow cytometry was performed to identify the different induced TNFR2 populations and their IFN-g production was determined. Data is representative of four donors. Kruskal–Wallis with Dunn’s multiple comparison test was used and error bars indicate SEM. Comparison of proliferation, mRNA and cytokine levels: *p < 0.05; **p < 0.01; ***p < 0.001.

To further analyze the function of induced CD25^hi^TNFR2^+^ T cells, we assessed their proliferative capacity. As shown in Figure 4A, induced CD25^hi^TNFR2^+^ T cells had a significantly higher proliferative capacity compared to the TNFR2^−^ T cells when re-stimulated with CD3/28 cross linking. Analyzing the cytokine production capacity of the sorted CD25^hi^TNFR2^+^ T cells, we found that intracellular IL-2 production in TNFR2^+^ cells were significantly higher compared to the TNFR2^−^ or TNFR2^int/−^ subsets (Figure 4B), suggesting a mechanism underlying both their increased proliferative capacity and ability to enhance effector T cell proliferation. The CD25^hi^TNFR2^+^ T cells also secreted significantly higher levels of IFN-g into the supernatant compared to the TNFR2^−^ or TNFR2^int/−^ subsets (Figure 4D), and interestingly, they also secreted IL-10, while the TNFR2^int/−^ and TNFR2^−^ cells did not secrete this cytokine (Figure 4C). IL-10 secretion, however, was present at a much lower concentration when compared to IFN-g and clearly insufficient to suppress proliferative responses. The phenotype of the induced CD25^hi^TNFR2^+^ T cells was further confirmed by mRNA expression level, determined using qPCR. Compared to TNFR2^int/−^ and TNFR2^−^ cells, the *in vitro* induced CD25^hi^TNFR2^+^ T cells expressed significantly higher mRNA level for IFN-g, IL-10 and the Th1 transcription factor, T-bet (Figures 4C,D). We also analyzed intracellular IFN-g production by these different induced TNFR2 populations from total PBMCs. Consistent with the mRNA levels and the cytokine levels present in the supernatant, we observed that the induced CD25^hi^TNFR2^+^ T cells have the highest intracellular production of IFN-g compared to the other induced populations, TNFR2^−^ and TNFR2^int/−^ T cells (Figure 4E).

## Discussion

In contrast to studies demonstrating effector T cells acquire only low levels of FOXP3 upon activation (8, 19), we find that induced CD25^hi^TNFR2^+^ T cells *in vitro* from CD4 T cells express high levels of FOXP3. Moreover, although it has been accepted as dogma that FOXP3 expression turns off IFN-g production (7, 8, 20), our results suggest that upon T cell activation, CD25^hi^TNFR2^+^ express not only IFN-g but also IL-2, IL-10, T-bet and thus these T cells identify a maximally active cytokine-producing subset. Although several studies have demonstrated that TNFR2 can be up-regulated on murine and human T cells upon activation (17, 21, 22), our study is the first to demonstrate that TNFR2 expression on human CD4^+^ T cells is concomitantly up-regulated with FOXP3 upon polyclonal TCR stimulation.

Despite high FOXP3 expression, which is a master regulatory gene enabling suppressive cell function (23), the role of induced CD25^hi^TNFR2^+^FOXP3^+^ T cells in the immune system may not necessarily be immune-suppressive. It is possible that the induced CD25^hi^TNFR2^+^ subset is a heterogeneous population containing both effector and T_regs_, however we have demonstrated here that the effector T cells clearly dominate in function in this induced subset. Moreover, the plasticity among T cells is a well-established phenomenon (24, 25) and hence it is not accurate to distinguish cells based merely on their phenotype without considering the nature of their induction.

**Figure A3:**
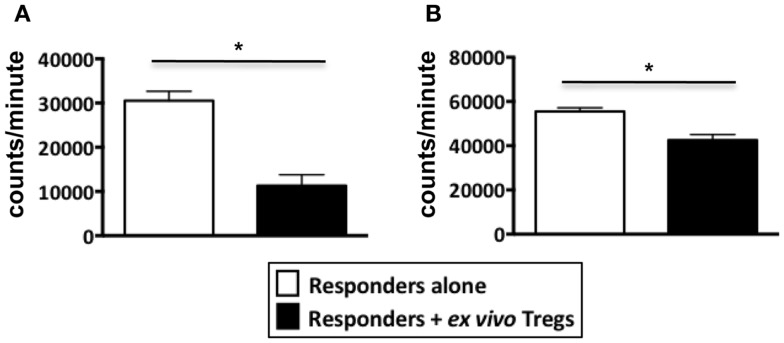
**Suppression assays performed using *ex vivo* T_regs_ from healthy donors**. PBMCs from healthy donors were stained with T_reg_ markers, CD4, CD25, and TNFR2 to sort for non-induced *ex vivo* T_regs_. T_regs_ were identified as either CD4^+^CD25^hi^TNFR2^+^ T cells or CD4^+^CD25^hi^TNFR2^−^ T cells. The sorted T_regs_ were added to autologous responders (CD4^+^ T cells) at a ratio of 1:1 and stimulated with anti-CD3/28 for 72 h in 96 well plates. **(A)** Represents a suppression assay using CD25^hi^TNFR2^+^ T cells as the T_reg_ population while **(B)** Represents a suppression assay using CD25^hi^TNFR2^−^ T cells as the T_reg_ population. Data shown here represents mean ± SD. Unpaired *t*-tests was used to determine statistical significance. *p < 0.05.

Our findings may seem to be contradictory to previous studies, which demonstrate that freshly isolated CD25^+^TNFR2^+^ T cells that express high levels of FOXP3 were maximally suppressive (10, 12). We do not believe a lack of suppression in the induced CD25^hi^TNFR2^+^ population was due to the difference in the suppression assay protocol employed as we have also demonstrated that *ex vivo* T_regs_ (both TNFR2^+^ and TNFR2^−^ T_reg_ subsets) are capable of suppressing responder T cell proliferation (Figure A3 in Appendix). However, this disparity in function between *ex vivo* and induced cells with a similar phenotype may be explained by the history of the cells, for instance, there could be functional differences between freshly isolated cells obtained from a balanced immune micro-environment and induced cells obtained as a result of polyclonal TCR stimulation of CD4 T cells. The potential plasticity of T cells, or how they may change phenotype and/or function in response to microenvironments, implies that the types of stimuli or culture conditions play a role in the phenotype or function of the induced T cells. We demonstrate here that *in vitro* stimulation of T cells with anti-CD3 and anti-CD28 results in several populations with varying effector functions, but none of the induced populations were suppressive in function.

Our results together with previous studies indicate that whereas freshly isolated peripheral blood CD25^+/hi^TNFR2^+^ T cells help maintain homeostasis, by preventing the activation of self-reactive cells in the absence of an active immune response, or after antigen clearance, induced CD25^hi^TNFR2^+^ T cells generated rapidly from circulating precursors by TCR stimulation in the absence of micro-environmental signals, would by contrast play a pivotal role in initiating responses against potential pathogens by maximally producing effector cytokines.

## Conflict of Interest Statement

The authors declare that the research was conducted in the absence of any commercial or financial relationships that could be construed as a potential conflict of interest.
